# *Architectural intelligence:* toward a spatial framework for interpreting microbiome-associated human disease

**DOI:** 10.1093/ismejo/wrag180

**Published:** 2026-07-12

**Authors:** Harikrishnan Parappalliyalil, Ananya Padmakumar, Debnath Ghosal, Eric C Reynolds

**Affiliations:** Oral Health Cooperative Research Centre, Melbourne Dental School, Bio21 Molecular Science and Biotechnology Institute, The University of Melbourne, Parkville, VIC 3010, Australia; Oral Health Cooperative Research Centre, Melbourne Dental School, Bio21 Molecular Science and Biotechnology Institute, The University of Melbourne, Parkville, VIC 3010, Australia; Department of Biochemistry and Pharmacology, Bio21 Molecular Science and Biotechnology Institute, The University of Melbourne, Parkville, VIC 3010, Australia; Oral Health Cooperative Research Centre, Melbourne Dental School, Bio21 Molecular Science and Biotechnology Institute, The University of Melbourne, Parkville, VIC 3010, Australia

**Keywords:** microbiome architecture, spatial configuration, ecological state

## Is there a missing spatial axis in microbiome-associated disease

Microbiome-associated diseases are still interpreted primarily through two dimensions: microbial composition and host response. Advances in sequencing have enabled high-resolution profiling of taxonomic and functional shifts across disease states, but these approaches treat microbial communities as homogenized populations. This assumes that abundance adequately represents ecological behavior. However, in host-associated systems, microbes exist within structured environments where spatial proximity influences the microbial interactions and functions, while microbial activity can in turn modify local spatial organization [1]. As a result, communities with similar compositions can exhibit fundamentally different ecological behaviors when their spatial organization differs. This limitation reflects a broader issue in how microbial communities are characterized. While spatial organization is widely recognized, particularly in biofilms, it remains largely descriptive and is rarely incorporated into analytical or diagnostic frameworks. Most studies continue to rely on compositional summaries, effectively decoupling structure from function and limiting the interpretation of microbial ecology in disease.

The distinction becomes clearer when considering what is meant by spatial structure. Biogeography describes the spatial distribution of taxa: where organisms are located. In contrast, microbial architecture includes not only distribution but also the relational organization of cells, including adjacency, clustering, layering, and the formation of gradients. These features influence how microbes interact, not just where they are positioned, while microbial interactions themselves can reshape spatial organization over time. If spatial organization is a defining property of host-associated microbial communities, the next question is what forms this organization takes *in situ*. Across systems, imaging of intact communities shows that microbial populations do not assemble randomly but instead adopt reproducible spatial architectures shaped by local environmental constraints and interaction networks.

The oral microbiome provides one of the best-characterized examples of this phenomenon. High-resolution imaging of intact dental plaque demonstrates that microbial taxa occupy defined micron-scale positions, forming structured consortia such as corncobs and radially organized “hedgehog” architectures [2,3]. These arrangements are not merely morphological but represent ecological configurations in which metabolic interactions, oxygen gradients, adhesion specificity, and spatial organization are reciprocally linked, collectively influencing taxon placement and community function [4]. In dental caries, spatial organization is directly linked to pathogenic function: EPS-rich, “rotund-shaped” microcolonies generate localized acidic niches that drive enamel demineralization, demonstrating that virulence can emerge from spatial configuration rather than composition alone [5].

Spatial organization can also emerge from underlying ecological interactions. For instance, *Aggregatibacter actinomycetemcomitans* positions itself at characteristic distances from *Streptococcus gordonii,* balancing access to cross-fed metabolites while avoiding inhibitory peroxide concentrations [6]. Such arrangements illustrate how metabolic interactions can shape architecture, reinforcing the reciprocal relationship between spatial organization and function. Similar feedbacks occur across microbial ecosystems, including soil aggregates and plant rhizospheres, where nutrient gradients, diffusion constraints and metabolite exchange contribute to spatial structuring and ecological specialization [7].

Similar organizational constraints are evident across other host-associated systems, although expressed through different structural forms. In mucus-dominated environments such as the gut and respiratory tract, microbial communities exist as non-attached aggregates or mucosa-associated layers. In the colon, a stratified mucus system separates host tissue from luminal microbes, while disease states such as colorectal cancer are associated with dense biofilm layers adherent to the epithelium [8,9]. In chronic respiratory infections, including cystic fibrosis, bacteria such as *Pseudomonas aeruginosa* persist as suspended aggregates within mucus, where spatial proximity and diffusion limitation contribute to oxygen gradients that are associated with antibiotic tolerance and immune evasion [10,11].

On the skin, microbial communities are organized as micron-scale, niche-confined clusters within follicles, glands, and pores, where local physicochemical conditions constrain growth and interaction. Rather than forming continuous biofilms, bacteria such as *Cutibacterium acnes* and *Staphylococcus spp*. persist as localized aggregates adapted to lipid-rich, low-oxygen environments [12]. Similarly, in the nasal cavity, microbial communities form discrete epithelial-associated clusters or mucus-embedded aggregates, reflecting constraints imposed by host adhesion, mucociliary clearance, and nutrient limitation.

**Figure 1:**
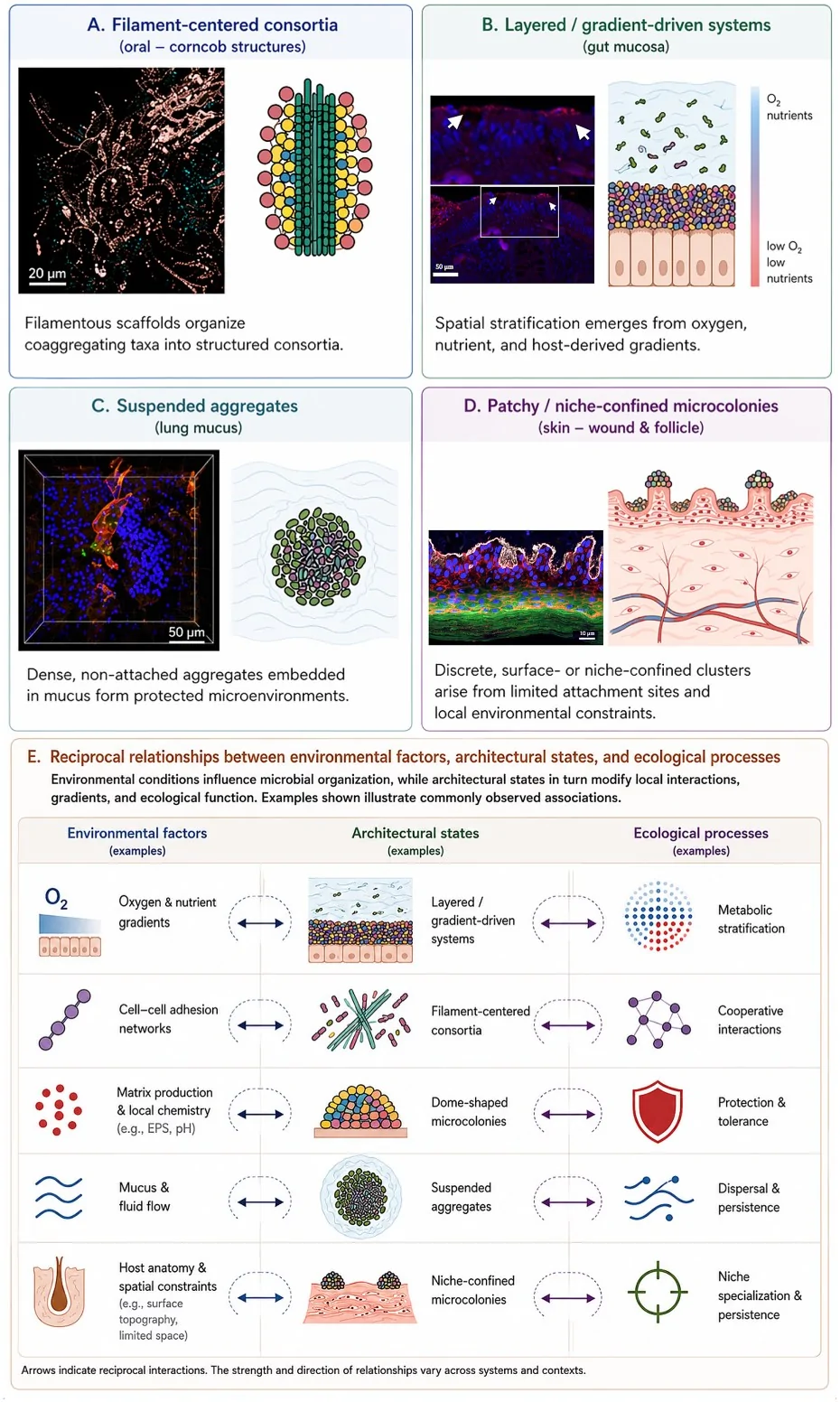
Spatial architectures of host-associated microbial communities. Host-associated microbial communities adopt reproducible spatial architectures that reflect local environmental constraints and interaction networks, shaping ecological functions. (A) Filament-centered consortia (oral). Filamentous scaffolds organize coaggregating taxa into spatially ordered consortia via specific adhesion interactions. (B) Layered / gradient-driven systems (gut mucosa). Oxygen, nutrients, and host-derived gradients generate spatial stratification and metabolic differentiation. (C) Suspended aggregates (mucus). Dense, non-attached aggregates embedded in mucus create diffusion-limited microenvironments that promote persistence and tolerance. (D) Patchy / niche-confined microcolonies (skin). Discrete clusters form at restricted attachment sites, shaped by host topology and physicochemical constraints. (E) Reciprocal relationships between environmental factors, architectural states, and ecological processes. Environmental factors interact with microbial communities to shape architectural states, which in turn influence ecological processes. The strength and direction of these relationships may vary across systems and contexts. All confocal micrographs adapted from [5, 9, 14, 15] (A–D) respectively, all licensed under CC BY 4.0.

Taken together, these observations indicate that spatial organization reflects recurrent ecological responses to environmental constraints [13]. Architecture both influences and emerges from microbial interactions and environmental conditions. It should therefore be viewed as both a product and a determinant of microbial ecology. Together, these observations converge on a central principle: microbial communities occupy discrete spatial configurations that reflect their ecological state. Rather than treating spatial organization as descriptive, these configurations can be interpreted as functionally meaningful entities. This suggests that microbiome-associated disease cannot be fully understood through microbial composition and host response alone. We therefore propose a tripartite framework in which microbial composition, host response, and spatial organization represent three complementary and interacting axes that define microbial ecology in disease. These recurrent spatial architectures across host-associated microbial systems are summarized in Fig.  1.

## Architectural states as ecological phenotypes

The recognition that microbial communities are spatially organized raises a critical question: how can this organization be systematically interpreted? We propose that recurrent spatial configurations observed across host-associated microbiomes can be formalized as *architectural states*: discrete, spatially defined ecological phenotypes that capture how microbial communities are organized and function *in situ*. Architectural states extend beyond spatial organization by describing the integrated configuration of a community, including the arrangement of taxa, their relative positioning, gradients, and interaction with host structures. Rather than focusing on which organisms are present, architectural states describe how those organisms are assembled into functional systems. Two communities with similar composition may therefore occupy different architectural states if their spatial configurations differ, leading to distinct ecological behaviors despite taxonomic similarity.

This distinction is particularly important in host-associated microbiomes, where spatial arrangement and interaction networks are closely coupled and continuously influence one another [10]. Cell positioning constrains access to nutrients, exposure to oxygen, metabolite exchange, and interaction with host immune factors, while these processes can subsequently reinforce or alter community organization [10,16,17]. As a result, architectural states encode functional potential by influencing which interactions are physically feasible, while the interactions themselves may contribute to the maintenance or emergence of architectural states. In this sense, they represent *ecological phenotypes*: emergent properties of microbial communities that arise from the combined effects of composition, environment, and spatial constraint. For architectural states to be analytically useful, they must be grounded in measurable features. We therefore distinguish *architectural biomarkers* as the quantifiable descriptors that define and differentiate these states (Box 1). Architectural biomarkers capture the relational properties including taxon-specific clustering, nearest-neighbor relationships, adjacency networks, radial or layered organization, aggregate size and density, matrix association, and proximity to host surfaces.

**Box 1:** What is an architectural biomarker?

An architectural biomarker is a quantitative descriptor derived from the spatial organization of microbial communities in intact biofilms. Unlike compositional biomarkers, which measure relative abundance, architectural biomarkers capture how microbial cells are arranged in space and how this organization correlates with ecological state in health and disease.
**Core properties:**
Spatially derived: Extracted from intact spatial imaging datasets that preserve community structure rather than homogenized samples. Feature-based: Represented as measurable spatial metrics, including cluster topology, radial or layered organization, nearest-neighbor adjacency networks and microhabitat occupancy. Ecologically interpretable: Reflects biological processes such as niche partitioning, metabolic stratification and cooperative assembly. Complementary to composition: Adds a structural dimension to taxonomic and host-derived biomarkers. Computationally extractable: Requires automated segmentation, spatial graph modeling, and interpretable machine learning for scalable implementation.
Architectural biomarkers therefore represent the measurable units of architectural states.

In this framework, architectural states and architectural biomarkers operate at different levels of abstraction. *Architectural states represent higher-order ecological configurations, such as layered biofilms, filament-associated consortia, dense aggregates, or niche-confined clusters. Architectural biomarkers, in contrast, represent the measurable features, for example, clustering coefficients, spatial autocorrelation, or host-distance gradients that define these configurations.* This enables spatial organization to be translated from a descriptive concept into a quantitative and comparable framework. Collectively, this approach allows microbial communities to be interpreted dynamically. Transitions between architectural states may occur during disease progression, environmental perturbation, or treatment. A community may shift from a spatially dispersed or host-compatible configuration to a dense, diffusion-limited, host-adjacent state associated with inflammation or persistence, even in the absence of major compositional change [18]. Conversely, therapeutic interventions may restore not only composition but also spatial organization, returning the community to a more stable architectural state. Architectural states therefore provide a framework for understanding microbial communities as dynamic ecological systems, rather than static taxonomic profiles.

**Figure 2:**
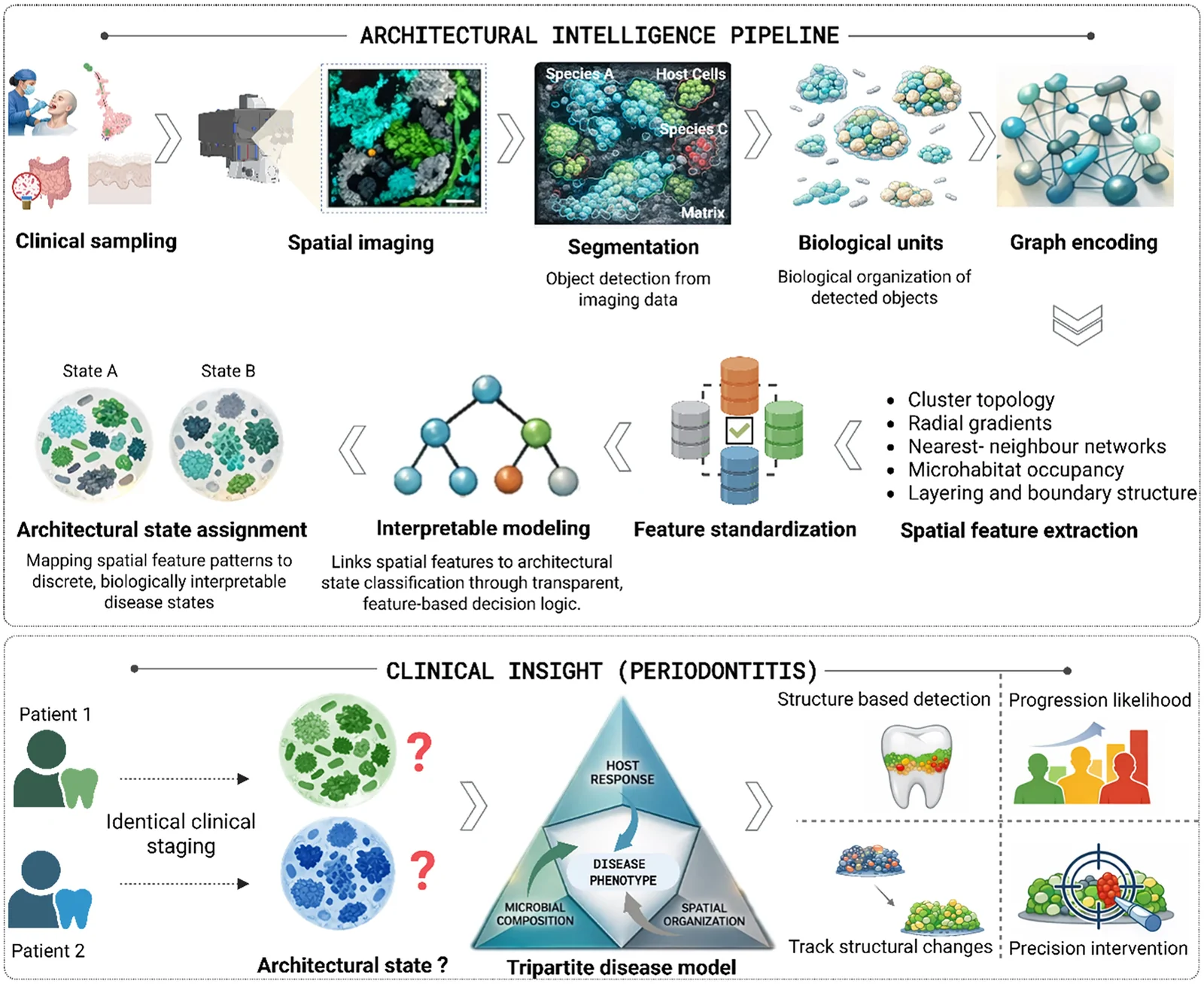
Architectural intelligence pipeline and clinical interpretation in periodontitis. Top: Schematic of the architectural intelligence workflow, from clinical sampling and spatial imaging to segmentation, graph-based encoding, spatial feature extraction, and architectural state assignment. Spatial features (architectural biomarkers) are standardized and integrated through interpretable modelling [19] to classify microbial communities into discrete architectural states. Bottom: Clinical application within the tripartite framework. Patients with similar clinical staging and microbial composition may exhibit distinct architectural states, leading to different disease trajectories. Integration of spatial organization with host response and composition enables structure-based diagnosis, tracking of disease progression, and precision intervention.

By defining architectural states as ecological phenotypes and architectural biomarkers as their measurable descriptors, spatial organization becomes an interpretable and testable dimension of microbiome science. This establishes the basis for a systematic framework in which spatial configuration can be quantified, compared across systems, and integrated with host response and composition. In doing so, it provides the conceptual foundation for *architectural intelligence*: the use of spatial organization as an additional interpretive and predictive dimension.

## Architectural intelligence: Operationalizing the spatial axis

The formulation of architectural states and architectural biomarkers provides a foundation for translating spatial organization into an operational framework (Fig.  2). We define *architectural intelligence as the systematic extraction, quantification, and interpretation of architectural biomarkers to infer ecological states, predict microbial behaviour and classify communities into biologically meaningful architectural states*. This framework leverages advances in spatial imaging and computational analysis to move from qualitative observation to quantitative inference. Intact microbial communities are first captured through high-resolution imaging [20], followed by automated segmentation to identify cells, taxa, and matrix components. These elements are then encoded into spatial representations, enabling the extraction of architectural biomarkers such as clustering patterns, adjacency networks, gradients, and host-interface relationships. Through feature selection and interpretable modelling, these spatial descriptors can be mapped to discrete architectural states, linking measurable structure to ecological phenotype [21]. Selection ensures that features derived from different samples, imaging conditions, or scales are normalized and directly comparable, while interpretable machine learning approaches, such as feature-weighted classification or graph-based models, identify the spatial features that most strongly define each state. This enables not only accurate classification, but also biological interpretation of which structural characteristics drive differences between ecological configurations.

Because microbial architecture can vary substantially across spatial scales, robust classification will require appropriate sampling strategies. Multiple fields of view, hierarchical image acquisition, and ecosystem-level replication may be necessary to distinguish localized microenvironments from broader ecosystem phenotypes. As with compositional profiling, representative sampling will be essential to ensure that assigned architectural states accurately reflect community-level organization.

Architectural intelligence operates within the tripartite framework, integrating spatial organization with microbial composition and host response. By resolving communities into architectural states, it enables distinctions that remain hidden under conventional metrics, allowing microbial systems with similar composition or clinical staging to be differentiated based on their interaction structure and ecological behavior. This has direct implications for diagnosis. If microbial behavior is governed by spatial configuration, then composition alone provides an incomplete representation of disease. Structure-informed classification enables the identification of ecological transitions before detectable shifts in abundance or host response, improving disease stratification and enabling earlier, more precise intervention.

More broadly, architectural intelligence reframes ecological interpretation. Rather than viewing dysbiosis as a change in community membership, it can be understood as a reorganization of interaction topology that reshapes how metabolites, signals, and resources flow through the system. In this view, microbial communities are defined not only by their composition, but by the spatial relationships that govern interaction and function. These insights extend to intervention. Treatments that target composition alone may fail when microbial behavior is governed by spatial configuration. Targeting microbial architecture, by disrupting dense aggregates, modifying matrix-associated structures, or restoring stable spatial states, offers a complementary strategy that addresses spatially mediated resistance and enables more precise, structure-informed interventions.

## Outstanding questions

- **Universality of microbial architecture**

Are spatial architectures conserved across individuals, niches and microbiomes, or are they context-specific and dynamic?

- **Causality versus consequence**

Do specific architecture drive disease processes, or do they emerge from host and environmental conditions?

- **Defining architectural units**

What constitutes a fundamental “architectural unit” in microbial communities, and how can these be standardized?

- **Scaling of organization**

How do microbial structures scale from microscale interactions to mesoscale architecture and macroscale function?

- **Temporal dynamics**

How stable are architectural states over time, and what governs transitions between health and disease-associated structures?

- **Integration with multi-omics**

How can spatial architecture be integrated with genomic and metabolomic data to generate unified ecological models?

- **Measurement and reproducibility**

What specifications are required to quantify architecture across imaging platforms and ensure reproducibility?

- **Interpretable modelling**

How can computational models remain biologically interpretable while capturing complex spatial relationships?

- **Clinical transition**

Can architectural biomarkers improve diagnosis, prognosis, or treatment stratification?

- **Perturbation and control**

Can microbial architecture be predictably manipulated to restore healthy states?

## Data Availability

Data sharing is not applicable to this article as no datasets were generated or analysed during the current study.

## References

[1] Nadell CD, Drescher K, Foster KR. Spatial structure, cooperation and competition in biofilms. *Nat Rev Microbiol* 2016;14:589–600. 10.1038/nrmicro.2016.84 27452230

[2] Morillo-Lopez V, Sjaarda A, Islam I, et al. Corncob structures in dental plaque reveal microhabitat taxon specificity. *Microbiome* 2022;10:145. 10.1186/s40168-022-01323-x PMC9446765 36064650

[3] Zijnge V, van Leeuwen MBM, Degener JE, et al. Oral biofilm architecture on natural teeth. *PLoS One* 2010;5:e9321. 10.1371/journal.pone.0009321 PMC2827546 20195365

[4] Borisy GG, Valm AM. Spatial scale in analysis of the dental plaque microbiome. *Periodontol.* 2021;2000:97–112. 10.1111/prd.12364 PMC8972407 33690940

[5] Kim D, Barraza JP, Arthur RA, et al. Spatial mapping of polymicrobial communities reveals a precise biogeography associated with human dental caries. *Proc Natl Acad Sci U S A* 2020;117:12375–86. 10.1073/pnas.1919099117 PMC7275741 32424080

[6] Stacy A, Everett J, Jorth P, et al. Bacterial fight-and-flight responses enhance virulence in a polymicrobial infection. *Proc Natl Acad Sci U S A* 2014;111:7819–24. 10.1073/pnas.1400586111 PMC4040543 24825893

[7] Bäcker M, Doekes HM, Garza DR, et al. Spatial structure: shaping the ecology and evolution of microbial communities. *FEMS Microbiol Rev* 2026;50:fuaf067. 10.1093/femsre/fuaf067 PMC12892361 41661138

[8] Johansson MEV, Larsson JMH, Hansson GC. The two mucus layers of colon are organized by the MUC2 mucin, whereas the outer layer is a legislator of host–microbial interactions. *Proc Natl Acad Sci* 2011;108:4659–65. 10.1073/pnas.1006451107 PMC3063600 20615996

[9] Dejea CM, Wick EC, Hechenbleikner EM, et al. Microbiota organization is a distinct feature of proximal colorectal cancers. *Proc Natl Acad Sci* 2014;111:18321–6. 10.1073/pnas.1406199111 PMC4280621 25489084

[10] Bjarnsholt T, Alhede M, Alhede M, et al. The *in vivo* biofilm. *Trends Microbiol* 2013;21:466–74. 10.1016/j.tim.2013.06.002 23827084

[11] Worlitzsch D, Tarran R, Ulrich M, et al. Effects of reduced mucus oxygen concentration in airway pseudomonas infections of cystic fibrosis patients. *J Clin Invest* 2002;109:317–25. 10.1172/JCI0213870 PMC150856 11827991

[12] Byrd AL, Belkaid Y, Segre JA. The human skin microbiome. *Nat Rev Microbiol* 2018;16:143–55. 10.1038/nrmicro.2017.157 29332945

[13] Berg G, Rybakova D, Fischer D, et al. Microbiome definition re-visited: old concepts and new challenges. *Microbiome* 2020;8:103. 10.1186/s40168-020-00875-0 PMC7329523 32605663

[14] DePas WH, Starwalt-Lee R, van Sambeek L, et al. Exposing the three-dimensional biogeography and metabolic states of pathogens in cystic fibrosis sputum via hydrogel embedding. *Clearing, and rRNA Labeling mBio* 2016;7:e00796-16. 10.1128/mBio.00796-16 PMC5040109 27677788

[15] Popov L, Kovalski J, Grandi G, et al. Three-dimensional human skin models to understand *Staphylococcus aureus* skin colonization and infection. *Front Immunol* 2014;5:41. 10.3389/fimmu.2014.00041 PMC3915142 24567733

[16] Flemming H-C, Wingender J, Szewzyk U, et al. Biofilms: an emergent form of bacterial life. *Nat Rev Microbiol* 2016;14:563–75. 10.1038/nrmicro.2016.94 27510863

[17] Moraïs S, Mizrahi I. Micro-scale spatial metagenomics opens a new era in microbiome ecology. *Trends Microbiol* 2026. 10.1016/j.tim.2026.03.005 PMC13460059 41934012

[18] Chu EK, Kilic O, Cho H, et al. Self-induced mechanical stress can trigger biofilm formation in uropathogenic Escherichia coli. *Nat Commun* 2018;9:4087. 10.1038/s41467-018-06552-z PMC6173693 30291231

[19] Wang J, Hashem I, Bhonsale S, et al. Individual-based modeling unravels spatial and social interactions in bacterial communities. *ISME J* 2025;19:wraf116. 10.1093/ismejo/wraf116 PMC12411854 40838740

[20] Shi H, Shi Q, Grodner B, et al. Highly multiplexed spatial mapping of microbial communities. *Nature* 2020;588:676–81. 10.1038/s41586-020-2983-4 PMC8050837 33268897

[21] Richardson M, Zhao S, Lin L, et al. SAMPL-seq reveals micron-scale spatial hubs in the human gut microbiome. *Nat Microbiol* 2025;10:527–40. 10.1038/s41564-024-01914-4 PMC12120799 39901058

